# The successful experimental induction of necrotic enteritis in chickens by *Clostridium perfringens*: a critical review

**DOI:** 10.1186/1297-9716-43-74

**Published:** 2012-10-26

**Authors:** Bahram Shojadoost, Andrew R Vince, John F Prescott

**Affiliations:** 1 Department of Clinical Sciences, Faculty of Veterinary Medicine, University of Tehran, Tehran, P.O. Box: 14155–6453, Iran; 2 Department of Pathobiology, University of Guelph, Guelph, Ontario, N1G 2W1, Canada

## Abstract

Necrotic enteritis (NE) is one of the most important enteric diseases in poultry and is a high cost to the industry worldwide. It is caused by avian-specific, Necrotic Enteritis Beta toxin (NetB)-producing, strains of *Clostridium perfringens* that also possess in common other virulence-associated genes. In Europe the disease incidence has increased since the ban on in-feed “growth promoting” antibiotics. Because of this, many recent studies of NE have focused on finding different ways to control the disease, and on understanding its pathogenesis. Frustratingly, reproduction of the disease has proven impossible for some researchers. This review describes and discusses factors known to be important in reproducing the disease experimentally, as well as other considerations in reproducing the disease. The critical bacterial factor is the use of virulent, *netB*-positive, strains; virulence can be enhanced by using *tpeL*- positive strains and by the use of young rather than old broth cultures to increase toxin expression. Intestinal damaging factors, notably the use of concurrent or preceding coccidial infection, or administration of coccidial vaccines, combined with *netB*-positive *C. perfringens* administration, can also be used to induce NE. Nutritional factors, particularly feeding high percentage of cereals containing non-starch polysaccharides (NSP) (wheat, rye, and barley) enhance disease by increasing digesta viscosity, mucus production and bacterial growth. Animal proteins, especially fish meal, enhance *C. perfringens* proliferation and toxin production. Other factors are discussed that may affect outcome but for which evidence of their importance is lacking. The review compares the different challenge approaches; depending on the aim of particular studies, the different critical factors can be adjusted to affect the severity of the lesions induced. A standardized scoring system is proposed for international adoption based on gross rather than histopathological lesions; if universally adopted this will allow better comparison between studies done by different researchers. Also a scoring system is provided to assist decisions on humane euthanasia of sick birds.

## Table of contents

1. Introduction

2. Different reasons to reproduce necrotic enteritis

3. Points to be considered in successful reproduction of necrotic enteritis

3.1. Nutritional factors

3.1.1. Feeding indigestible non-starch polysaccharides

3.1.2. Feeding large amounts of animal protein (fish meal)

3.2. Role of coccidia

3.3. The role of immunosuppression in experimental necrotic enteritis

3.4. Bacteriological aspects

3.4.1. Critical virulence features of *C. Perfringens* strains involved in necrotic enteritis in chickens, and in reproducing the disease

3.4.2. Preparation of *C. perfringens* for challenge

3.4.2.1. Type of culture media

3.4.2.2. Incubation time

3.4.2.3. Amount of bacteria for challenge

3.5. Challenge methods

4. Other considerations

5. Lesion scoring systems

5.1. Gross lesions of necrotic enteritis

5.2. Different scoring systems

6. Reproduction of clinical and subclinical necrotic enteritis

7. Determining performance parameters (Weight gain, Feed intake, feed conversion ratio = FCR)

8. Welfare considerations

9. Conclusions

10. Competing interests

11. Authors’ contributions

12. Acknowledgements

13. References

## 1. Introduction

Necrotic enteritis (NE) in chickens, first reported by Parish [1], is an enteric disease caused by *C. perfringens*, a Gram-positive anaerobic spore-forming, rod-shaped bacterium [2]. According to the current classification, *C. perfringens* has five toxinogenic types (A, B, C, D, E), which are differentiated according to the production of four different major toxins (Alpha, Beta, Epsilon, Iota) [3]. The discovery in recent years of new toxins (Beta2, NetB, TpeL) in *C. perfringens* shows the need for an enhanced classification scheme. NE is caused by type A isolates [3] and rarely by type C isolates [4,5]. The recently discovered new toxin, NetB, is crucial for development of the disease [6,7]. Keyburn et al.’s [6] seminal discovery of the crucial role of the pore-forming toxin NetB led to the subsequent characterization of three pathogenicity loci (PAL) that are characteristic of NE isolates [8]. Two PAL (NELoc1, NELoc 3) are plasmid-encoded, usually on different plasmids [8]. Two plasmids on which these PAL are found have recently been fully sequenced [9]. NE isolates belong to two major lineages or clones [10], suggesting that these lineages have adapted to cause NE in chickens.

The intestinal number of *C. perfringens* in healthy and in NE-affected birds are different. The *C. perfringens* population is found to be normally less than l0^2^ to 10^4^ colony-forming units (CFU) per g of the intestinal contents in the small intestine of healthy chickens compared to 10^7^ - 10^9^ CFU/g in diseased birds [11].

NE occurs in broilers aged between two and six weeks [4,12]. Mortality can reach 1% per day with a total mortality of 10-40% [13]. Clinical signs include depression, dehydration, diarrhea, ruffled feathers and lower feed intake [4]. The gross lesions of the small intestine range from thin and friable walls to frank and extensive necrotic lesions [12]. Two forms of the disease are described, clinical and subclinical [4,7,14]. The clinical form appears with the clinical signs and mortality noted above. The subclinical form presents as poor performance (reduced growth, reduced feed efficiency) without mortality. This form of the disease can be diagnosed by reduced feed conversion, by gross lesions in the small intestine and by bacteriology [14]. Most of the economic losses due to NE are related to the subclinical form and the high cost of preventing the disease with antibiotics.

Antibacterial drugs are commonly used to prevent or control the disease. In recent years, the European Union has banned the use of in-feed antimicrobials or growth promoters, leading to an increase in disease outbreaks in broiler flocks in European countries [15,16]. Globally, the economical impact of the disease is estimated at US$ 2 billion year through mortalities and poor performance and the cost of prevention and treatment [15,17]. In EU countries, the profit of severely NE affected broiler flocks has been 33% less than flocks with low level of the disease [17]. The variables affected the significant economic cost of subclinical NE have been estimated [18].

In recent years there has been an explosion of interest in understanding the pathogenesis of NE and investigating how it can be prevented by approaches other than the use of antibiotics. Many of these studies necessitate experimental induction of the disease. However, published and anecdotal reports attest to the difficulty that some workers have experienced in reproduction of the disease [16,19,20]. It is the purpose of this review to summarize the different approaches used to reproduce NE, to identify factors that are critical to successful production of experimental disease, and to highlight areas of uncertainty that require further investigation. The purpose of the review is to assist researchers to understand NE so that their experimental procedures are based on an evidence-based and logical approach.

**Figure 1:**
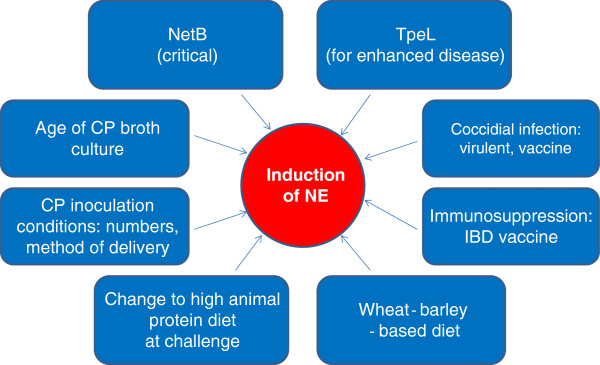
**Critical factors influencing the development of necrotic enteritis.** Summary of different factors important for the successful reproduction of necrotic enteritis; the most critical factor is presence of *netB* and the *netB* plasmid, but other factors summarized in the figure all affect the outcome of experimental infection. They can be manipulated by researchers to vary the severity of the disease produced and the outcome desired.

Necrotic enteritis is a complex and multi-factorial disease. The factors affecting development of the disease in the field were well summarized by Williams [21], unfortunately just before the discovery of NetB. Figure 1 is a simplified diagram, extending that of William’s earlier figure, which summarizes some of the critical factors influencing the development of the disease.

## 2. Different reasons to reproduce necrotic enteritis

Different researchers have different reasons for reproducing necrotic enteritis, which will impact the design of studies as well as the severity of disease that they wish to produce. Reasons to reproduce the disease have included comparison of antimicrobial drugs [22-27], vaccines [28-31], assessment of virulence determinants [6,32], or assessment of pathological processes [19]. Others have studied the effect of different predisposing factors including nutritional components on the severity of NE [2,33-37], the effects of probiotics and prebiotics [23], or different experimental models of disease [38-41]. Design considerations will also include the need for control groups and the need for isolation facilities to ensure lack of spread of infection between control and infected birds. The novice researcher is advised to perform a small scale pilot study both to confirm their ability to reproduce the disease and to identify the different effects of variables discussed below that affect the severity of the disease that they wish to produce. It would be anticipated that production of severe experimental disease would mask some of the beneficial effects of interventions such as feed components or immunization.

## 3. Points to be considered in successful reproduction of necrotic enteritis

### 3.1. Nutritional factors

#### 3.1.1. Feeding indigestible non-starch polysaccharides

Several studies have shown that feeds (barley, rye, wheat) containing high amount of water-soluble indigestible non-starch polysaccharide (NSP), such as β-glucans or arabinoxylans, increase the viscosity of the digesta and predispose chickens to NE [13,14,42,43]. The higher viscosity of digesta in diets containing wheat or barley leads to prolonged transit time in the intestine, which may be responsible for the direct correlation between intestinal viscosity and clostridial counts [42]. However, equally or more importantly, NSP also interact with glycoproteins on the epithelial surface to increase mucin production [44]. *C. perfringens* has an “arsenal” of up to 56 glycoside hydrolases directed at the muco-oligosacharides prominent in the mucosal layers of the intestine [45,46]. The two prominent chitinase genes present on the major pathogenicity locus (NELoc1) of NE isolates are speculated to have mucin degradation functions [8].

In a *C. perfringens* challenge model, mortalities in birds fed a supernatant of digested maize ranged from 0-12%, whereas birds that received barley, rye or wheat showed mortality of 26-35% [47]. Mortality due to NE in coccidiosis-challenged chicks with a corn-based diet was also less than mortality in a wheat-based diet [48]. The number of *C. perfringens* in the intestine in corn-based diet fed broilers were 1.2-1.5 log_10_ colony-forming units/g lower than in the birds fed 50% rye in their feed [49]. Others have also reported the increased incidence of NE associated with wheat or barley diets [14,50]. Since NSPs are not well digested they reach the lower intestinal tract and alter its environment [51,52].

The size of the feed particles has been shown to affect the number of *C. perfringens* in the intestine. As expected, highly ground feed allows *C. perfringens* to proliferate faster and to larger numbers than coarse ground feed [48,53], and can predispose birds to NE [48,54].

Other nutritional factors can impact the severity of NE. For example, trypsin inhibitors in non-toasted soya bean based ration increased the severity of NE lesions, in direct proportion to the quantity of soya bean in the feed [55]. Trypsin inhibition is well established as a predisposing factor to enteric disease caused by *C. perfringens*, since trypsin in the small intestine destroys *C. perfringens* toxins [4].

Researchers should ensure that feed of experimental birds contains neither antibiotics nor anti-coccidials, recognizing that the latter may also have antibacterial properties.

#### 3.1.2. Feeding large amounts of animal protein (fish meal)

A characteristic of *C. perfringens* identified through genome sequencing is the inability of this “anaerobic flesh eater” to synthesize the majority of amino acids; *C. perfringens* rapidly breakdown tissues through the extraordinary array of hydrolytic enzymes that it produces [56]. Large amounts of animal-origin protein in the diet predispose poultry to NE [21,50,57]. The presence of high crude protein concentration and some amino acids are related to commencement of *C. perfringens* overgrowth and production of alpha toxin [58]. Glycine is among the amino acids that stimulate growth and production of alpha toxin [59,60] and is positively correlated with the number of *C. perfringens* in the intestine [61]. Therefore the glycine content of the diet may be important to predispose birds to NE. The level of *C. perfringens* has been found highest with the greater amount of animal protein (40% crude protein/feed) and lowest in plant-source protein diets fed to chicks [19,62]. Large amounts of fish meal have been associated with *C. perfringens* proliferation and the occurrence of NE [63]. The amounts of glycine and methionine, which increase *C. perfringens* proliferation and alpha toxin concentrations in vitro, are higher than other amino acids in fish meal protein-based diets [60]. Although there has been no study of the effect of diet specifically on NetB toxin production, both the *netB* gene as well as the internalin gene adjacent to it on NELoc1 have VirR boxes [8,64], suggesting that, like alpha toxin, their expression is under control of the VirR-VirS regulatory system that controls virulence gene expression in this organism. It is therefore likely that conditions that increase alpha toxin also increase the critically important NetB.

Having a diet with, or changing the diet to one with high protein before the time of challenge seems to enhance the severity of NE although this effect has not been critically evaluated in detail, and comparisons are difficult because of use of different scoring systems. Changing the diet to a high protein diet before *C. perfringens* challenge increased the severity of induced NE, but mixing the diet with 30-50% fish meal has been done either on the same day [65], one day after [41], or seven [24,26,65,66] days before *C. perfringens* challenge. In one study, turkey feed (28% protein) was mixed with 50% fish meal and given to chicks from 1–13 days of age, which resulted in 12% mortality and 65% of the birds showing NE lesions when the chicks were challenged with *C. perfringens* at 14 days of age [22]. Consumption of diets containing lower energy: protein ratios not only leads to increased feed intake and higher nitrogen content of the digesta and feces [13] but can also lead to an enhanced substrate for *C. perfringens*[67,68]. It seems that feeding birds with a high protein diet for a longer period of time is better in reproducing more severe NE, but details of timing have not been determined. The high protein ration should be present at the time of challenge.

### 3.2. Role of coccidia

Coccidiosis is an enteric parasitic disease caused in poultry by various *Eimeria* spp. [21]. Some (*E. brunetti*, *E. maxima*, *E. necatrix*, *E. tenella*) produce more severe disease than others (*E. acervulina*, *E. mitis*, *E. praecox*) [21]. Damage to the epithelium caused by coccidia is a major predisposing factor for NE, allowing *C. perfringens* to replicate rapidly and produce toxin [69], probably because leakage of proteins including plasma into the lumen of the gut during *Eimeria* infection provides the protein-rich nutrient substrates favorable to *C. perfringens* proliferation and toxin production [16]. The mucogenesis induced by coccidial infection may also be important [70].

For these reasons, *Eimeria* spp. have often been used in conjunction with *C. perfringens* to induce NE experimentally. Since NE is a disease of small intestine, *E. acervulina*, *E. maxima* or *E. necatrix* are the most suitable species [2,15,23,40,71]. There are differences in opinion as to whether the more virulent *Eimeria*, such as *E. necatrix* are better than the less virulent *E. acervulina* choices for this purpose [2,72]. To obtain the effect of coccidial challenge, damage to the epithelium should occur before challenge with *C. perfringens*[43]. Table 1 summarizes the timing and the effect of different *Eimeria* spp. in conjunction with the timing of *C. perfringens* challenge. *Eimeria* vaccines have also been used to enhance the effect of *C. perfringens* challenge [20,41].

**Table 1:** Effect of coccidial challenge on severity of experimental necrotic enteritis

**Researchers**	***Eimeria *spp.**	**Age of coccidia challenge (day)**	**Age of *C. perfringens *challenge (day)**	**% Mortality**	
				**With coccidia**	**Without coccidia**
[71]	*E. necatrix*	11	15	28.3	16
[71]	*E. acervulina*	13	15	53.3	16
[70]	*E. acervulina E. maxima*	14	18, 19, 20	7.6	2.8
[2]	*E. necatrix*	4	5, 6, 7, 8, 9	22.5	0
[41]	Paracox-8 (Vaccine)	19	18, 19, 20, 21	0^1^	0
[41]	*E. maxima*	20	19, 20, 21, 22	0^1^	0
[41]	Paracox-8 (Vaccine)	16	19, 20, 21, 22	0^1^	0
[20]	Paracox-5 (vaccine)	20	19, 20, 21, 22	0^1^	0
[84]	Paracox-8 (Vaccine)	10	9, 10, 11, 12	0^1^	0^1^
[84]	Paracox-8 (Vaccine)	18	17, 18, 19, 20	0^1^	0^1^

^1^Sub-clinical necrotic enteritis (No mortality).

The time of administration of the coccidial vaccine or of virulent *Eimeria* is critical, and should be not more than 4–5 days before the *C. perfringens* challenge so that the coccidia-induced intestinal damage coincides with bacterial challenge [21,40]. When the *C. perfringens* challenge lasted for 4 days, no significant difference was observed in the severity of lesions in birds receiving coccidial vaccine 3 days before or one day after the onset of *C. perfringens* challenge [20,41].

The dose of *Eimeria* is also important; successful NE disease has been induced by 2 × 10^4^*E. necatrix* or 2–5 × 10^4^*E. maxima*[2,41,70]. Numbers above these may be fatal. For less pathogenic species such as *E. acervulina*, higher doses (7.5 × 10^4^ up to 5 × 10^5^) have been used [70,71]. Attenuated coccidial vaccines, which as commercial products may be more accessible for some researchers, have been used at 10-times recommended vaccination doses [20,41], since lower doses of the vaccine may not be as efficacious.

When reproducing NE, the purpose of the study will determine which combination of the *C. perfringens* and *Eimeria* spp. or *Eimeria* vaccines should be used, for example whether the intention is to produce severe or mild disease, or whether the intention is to test *C. perfringens* vaccines. The studies should be designed to include controls of *Eimeria* alone, *C. perfringens* alone, and combined *Eimeri*a and *C. perfringens*. The dose and species or type of coccidal challenge should be chosen with care, depending on the purpose of reproducing NE, since use of coccidia can readily result in severe disease. For example, when testing a vaccine, or a specific drug against NE, it is probably better to reproduce the NE without the help of virulent coccidial challenge, or to use attenuated coccidial vaccines.

An unresolved question is whether, if coccidia are used to enhance the disease process, it is a requirement for the *C. perfringens* to contain *netB*; it seems highly likely, but requires to be demonstrated.

### 3.3. The role of immunosuppression in experimental necrotic enteritis

Immunosuppressed chickens are more likely to develop necrotic enteritis, so that some researchers have used methods to induce immunosuppression. These methods mostly use Infectious Bursal Disease vaccine (IBD) [20,33,34,41]. It has resulted in a significant increase in NE lesions [33]. This has been done by administering a usual dose of IBD vaccine with a medium pathogenicity (intermediate class) [20,41], or 10 times the dose of an IBD vaccine with relatively higher pathogenicity (intermediate plus) [73]. The lesion scores are higher when IBD vaccine is used, even with a normal dose of an intermediate class IBD vaccine [41].

Stressful conditions may decrease immunity and predispose to NE [13]. Increasing stocking density, as a “stressful” measure, combined with IBD vaccine, was used in an experimental model of NE [73], but was not evaluated independently of IBD. Welfare considerations preclude deliberately “stressing” chickens. As evident from this review, there are numerous other factors that can be used to induce disease reliably. Use of immunosuppression as part of the induction of NE is inappropriate if vaccines are being evaluated.

### 3.4. Bacteriological aspects

#### 3.4.1. Critical virulence features of C. perfringens strains involved in NE in chickens, and in reproducing the disease

*Clostridum perfringens* is notorious for its ability to produce a wide variety of toxins. Although for many years alpha toxin, a chromosomally-encoded zinc metalloenzyme with lecithinase and sphingomyelinase activity [43], was thought to be the major virulence factor in NE [72,74], the early histological changes in NE are not consistent with the sphingomyelinase or phospholipase C activities of alpha-toxin [19]. A critical study by Keyburn et al. [32], showed that a *cpa* mutant, which was unable to produce alpha toxin, was still able to produce the disease. The final breakthrough in understanding NE was the demonstration by the same workers of a novel pore-forming toxin, NetB, which was shown to be essential in producing disease [6]. NetB has homology to the pore-forming beta toxin of *C. perfringens*, as well as to the alpha hemolysin and gamma toxin of *Staphylococcus aureus*[7]. The *netB* gene is mostly found in isolates from NE outbreaks, and is relatively uncommon in isolates from healthy birds [7,75-78]. In a chicken experimental model assessing the virulence of 10 *C. perfringens* isolates from several sources, including cattle, normal chickens, humans, soil and swine, Cooper et al. [79] found that only the *netB*-positive isolate from a field case of NE was able to cause disease.

The *netB* gene is associated with a 42 kb pathogenicity locus (NELoc-1), which is on a 85 kb plasmid, as well as with another possibly less important plasmid-associated locus, NELoc3, present on a separate plasmid, and with a chromosomally-located pathogenicity locus, NELoc2 [8,9]. It seems very likely that the difficulty that many workers have experienced in reproducing disease can be attributed to the loss of the entire virulence plasmid containing the *netB* pathogenicity locus. It is however insufficient simply to use *C. perfringens* isolated from NE lesions to try to reproduce NE, since not all these may contain *netB*[76]. Recent multilocus sequence typing (MLST) studies have also identified two prevalent clonal groups among NE-associated isolates [10,75], suggesting that not just the virulence plasmids but also specific chromosomal genes (i.e., the bacterial host background) may also be important in NE pathogenesis or possibly for maintenance of the plasmids.

*TpeL,* a member of Large Clostridial Toxins (LCT) family [80], is present in some type A NE isolates [75]. There is evidence that *netB*-positive strains that are also *tpeL*-positive cause more severe disease than strains that lack *tpeL*[81].

In summary, emerging understanding of NE is that the strain and particularly the virulence plasmids of NE isolates are critical in producing disease, with *netB* being the only virulence factor to date shown to be essential in producing disease. Severity of disease may vary with the presence of other virulence determinants, of which the best implicated accessory toxin is *tpeL*. Because of the complexity of virulence regulation in *C. perfringens*, spontaneous mutations in VirR-VirS or other regulatory genes may affect virulence. It would therefore be prudent before embarking on the expense of large-scale NE studies for researchers to compare and confirm the virulence of potential *netB*-positive challenge strains in a pilot study. If more severe disease is desired, it is recommended to obtain a strain possessing *tpeL*. Because of the danger of loss of virulence plasmids, researchers attempting to reproduce NE should continuously maintain a “paranoid” approach to confirming throughout their work (using PCR) that the experimental strain has not lost the *netB* gene (and therefore the *netB* plasmid). The PCR should be done on individual colonies, not as a “sweep” of a blood plate, since PCR is so sensitive that researchers may not recognize through “sweeps” of a plate that the strain is gradually losing plasmids as it is subcultured. Maintaining a frozen stock of the virulent strain will ensure against loss of plasmid during subculture. A specific strain designation should always be given to the challenge strain, which should be made available to other researchers on request.

#### 3.4.2. Preparation of clostridium perfringens for challenge

##### 3.4.2.1. Type of culture media

Several different anaerobic culture media and different incubation times have been used to reproduce NE. The choice depends on both convenience and cost, so that fluid thioglycolate medium (with dextrose) (FTG) is the most common culture medium used for challenge purposes [19,33-35,38,39,47]. Adding peptone and starch to this medium increased the level of alpha toxin production [72], although as noted it is NetB not alpha toxin that is critically important in NE. Others have used cooked meat medium (CMM) [36,60] or brain heart infusion broth (BHI) [82-84], but CMM is expensive and BHI does not have reducing agents in it, so that it needs to be cultured anaerobically which will likely be inconvenient. FTG broth has the advantage that it can be cultured aerobically. Many researchers cultured the *C. perfringens* challenge strain initially in fluid CMM and then subsequently inoculate this CMM “stock” into FTG [12,22,65,66,79,85-87]. This system is mostly used for convenience but partly for historical reasons. The FTG is used as the challenge inoculum.

##### 3.4.2.2. Incubation time

Younger (15 h) FTG cultures produce more severe disease than 24 h cultures [28,38] probably because of greater production of toxin(s) at this time. For example, 15 h broth culture increased lesion scores by over 50% compared to 24 h cultures [28]. It is also likely that older, stationary, phase cultures will produce proteases that degrade NetB and other toxins. Logistically, however, for researchers who challenge birds twice a day with broth, it may be difficult to prepare a 15 h culture for the afternoon challenge since they would need to start FTG incubation of their challenge strain at midnight. If researchers want to produce less severe disease, then an afternoon feeding with a 24-h culture can be used.

Successful challenge using *C. perfringens* alone depends on initiating intestinal damage by preformed toxin rather than by toxin produced in the intestine [72]. Therefore whole broth cultures (which contain pre formed toxins) and not just vegetative *C. perfringens* cells should always be used.

##### 3.4.2.3. Amount of bacteria for challenge

The amount of *C. perfringens* used for challenge is important for successful challenge. It is normally between 10^7^ to 10^9^ CFU/mL [33,34,60]. A 15-h overnight FTG broth culture inoculated with a 3% (v/v) overnight CMM culture and incubated at 37°C will contain over 10^8^*C. perfringens*/mL.

### 3.5. Challenge methods

One successful approach that has commonly been used is to present the challenge strain to birds through feed, inoculated at a ratio of 1.25-1.5 fluid FTG: feed (v/w). Birds are usually initially starved overnight so that they will eat the first batch of infected feed ravenously. Feed is usually prepared fresh twice daily (morning and evening). The period of feeding contaminated feed has varied from 1–5 days, with birds being euthanized on the day of or after the final feed [22,79,86]. On average, researchers challenging birds through the feed will challenge for 3 or 4 days and euthanize on day 4 or 5 (the first day after the last challenge). The time of exposure is important, since 5 days of challenge induced more than twice the mortality of one day challenge [38]. Infected feed should always be available to birds. The advantage of challenging birds through feed is that it does not involve handling birds individually. The disadvantage is that it involves handling and incubating large volumes of media, which can smell unpleasant because of production of hydrogen sulfide, requires often large number of flasks which logistically may be difficult to autoclave, and is expensive.

An alternative to infecting birds through large volumes of feed is to infect birds individually by crop gavage with broth cultures. This has involved inoculation of 3 mL of 10^5^-10^7^ colony-forming units twice daily for three consecutive days [33-35] or 1 mL of 1–4 × 10^8^ CFU/mL for 4 days or 7 days [19,20,41]. The method is combined with some NE risk factors such as coccidial challenge or IBD vaccination. For example, no lesions were detected using oral *C. perfringens* challenge without coccidial challenge [41]. McReynolds et al. [33] used IBD vaccine as an immunosuppressant followed by oral *C. perfringens* challenge, and this resulted to a significant increase in NE lesions comparing to the birds that just received *C. perfringens* challenge alone. However, as noted earlier, numerous studies have successfully used broth culture mixed with feed without using coccidial or immunosuppressing predisposition. In conclusion, researchers need to decide on the approach (*C. perfringens* alone in feed; coccidial or IBD immunosuppression, followed by *C. perfringens* given by gavage or in feed) that best fits their facilities, ability to handle birds, the severity of the disease that they wish to produce, and other considerations. Small-scale pilot studies using different variables should establish the conditions best fitted for their study.

## 4. Other considerations

Most studies use broiler chickens, usually of mixed sex. It is possible that male birds, which eat more, may be more susceptible, as are male turkeys in field cases [62]. Immunization of the parent flock has shown to reduce the NE lesions and mortality [88] so chicks should not be from hens vaccinated with *C. perfringens* vaccines. Challenge at 2–3 weeks of age should ensure low maternally-derived antibodies in chicks from unvaccinated hens.

## 5. Lesion scoring systems

### 5.1. Gross lesions of necrotic enteritis

**Figure 2:**
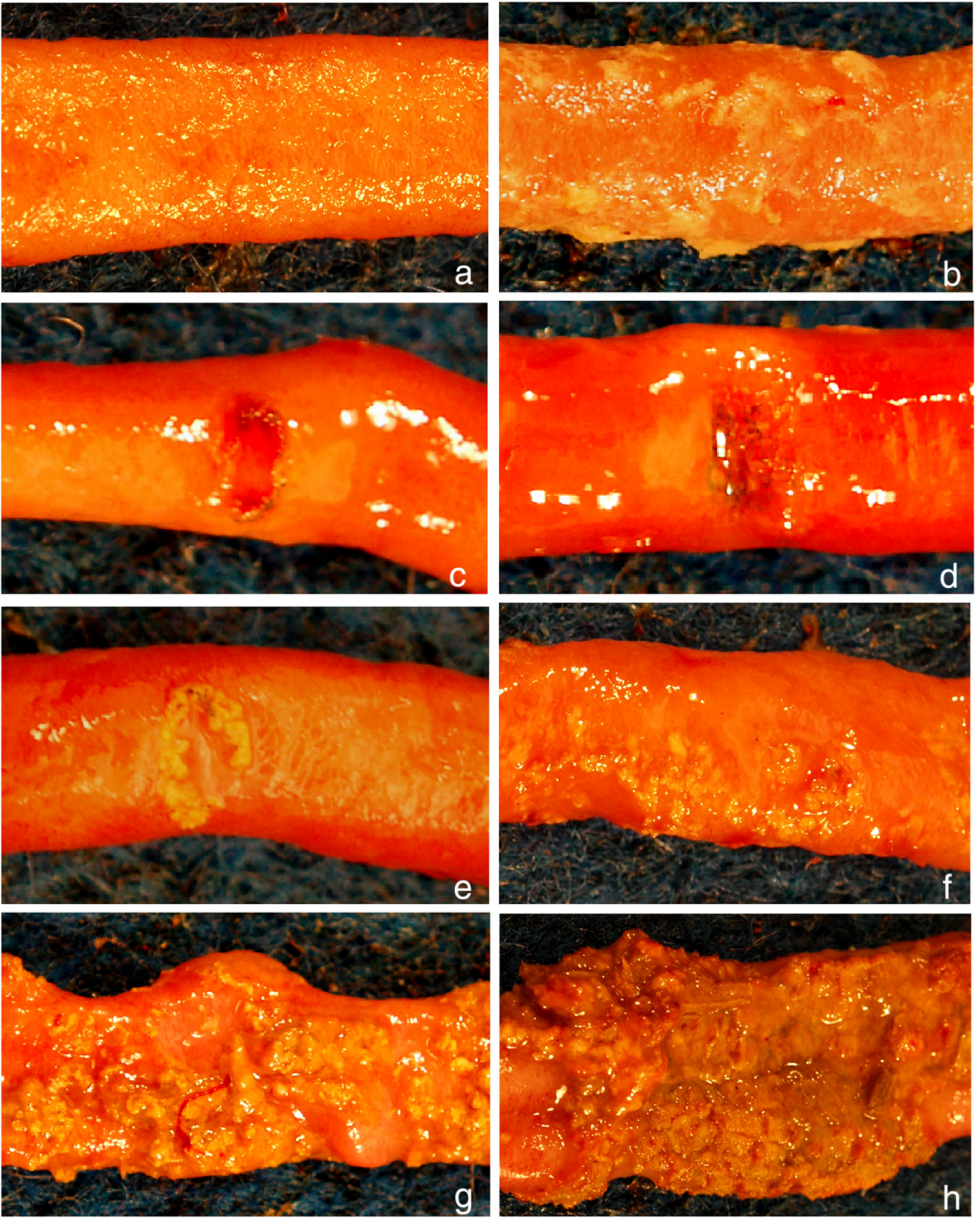
**Different lesions of necrotic enteritis in chickens, used to illustrate the scoring system (Table**2**).a**: Necrotic enteritis score 0, everted jejunal segment. No gross lesions are present. **b**: Necrotic enteritis score 1, everted jejunal segment. There are no obvious ulcers in the mucosa, but the entire mucosal surface is covered with a layer of loosely adherent fibrin. **c**: Necrotic enteritis score 2–4, everted jejunal segment. There is an excavated ulcer of the mucosa with acute, bright red hemorrhage within the ulcer bed and scant crusting of fibrin around the periphery. **d**: Necrotic enteritis score 2–4, everted jejunal segment. There is an excavated ulcer of the mucosa with dark green-black pigment within the ulcer bed and scant crusting of fibrin over the surface. **e**-**f**: Necrotic enteritis score 2–4, everted jejunal segments. There are excavated ulcers of the mucosae, the periphery of which are covered by thick, tightly-adherent layers of fibrin, necrotic tissue, and inflammatory cells. **g**-**h**: Necrotic enteritis score 5–6, everted jejunal segment. The mucosae are covered by large, confluent plaques of fibrin, necrotic tissue, and inflammatory cells (**g**) to the point where they extend over broad regions of the intestinal mucosa (**h**).

The gross small intestinal lesions of necrotic enteritis comprise a spectrum of mucosal erosion-to-ulceration that vary in both extent and in individual character and severity. In many animals, individual lesions of various characters may be identified. Normally, the mucosal surface of the intestinal tract should be smooth and shiny, reflecting an intact mucosal epithelial barrier (Figure 2a). With erosion and ulceration one may see a complex of subtle mucosal dullness (reflecting fibrin exudation; Figure 2b), cavitation/ulceration (Figures 2c-e), acute hemorrhage (seen as localized intense reddening, often co-localized to areas of cavitation/ulceration, occasionally with frank blood clots in the intestinal lumen; Figure 2c), subacute hemorrhage (seen as localized green-black mucosal pigmentation, often co-localized to areas of cavitation/ulceration; Figure 2d), and the formation of individual mucosal fibrin plaques that cannot be removed with a finger (Figure 2f). The disease process progresses, with or without obvious cavitation, to produce the large-scale accumulation of fibrin, necrotic tissue debris, and inflammatory cells that coalesce to form the suffusive extensive mat of tenacious exudate on the mucosal surface (Figures 2g-h) that is typical of field cases of birds dying of necrotic enteritis.

### 5.2. Different scoring systems

Following humane euthanasia, for example by overdose of carbon dioxide, birds are necropsied and the small intestine examined for gross pathological lesions. It is important to assess the entire length of the small intestine, since lesions may be present in the upper duo denum that are not found elsewhere. Most lesions will however be in the jejunum. A variety of systems for scoring NE gross lesions have been used, using scales that have varied from 0–3 [31,41,70], 0–4 [12,22,26,33,79,87] to 0 – 6 [6,20,32,65]. A serious drawback of this variability, and of the different criteria used even within these scoring systems, is that it is impossible to compare studies that use different scoring systems. International adoption of a common standard system will considerably ease comparison between studies, including comparison of different control methods. One difficulty however of adopting a common scoring system is that there is variation in the gross lesions of NE, described above, which anecdotally may be exacerbated by the effects of different interventions, such as for example immunization of birds with different antigens.

An ideal scoring system should encompass the severity of the disease produced experimentally, have a reasonably wide range for purposes of statistical analysis, be simple so that large numbers of birds can be examined in a reasonable time, and be reproducible between observers. The scoring system that best approximates these criteria is the six-point system of Keyburn et al. [32]. We therefore propose that this system, with slight modification (Table 2), becomes the international standard for experimental NE studies. The modification proposed is that the “1” includes the presence of non-adherent fibrin in affected intestines (Figure 2b) as an additional alternative to “thin walled and friable intestine”. It is recommended that researchers include an experienced pathologist in evaluating lesions of NE, that the lesions be scored “blind”, and that at least two people work together to score the lesions. Figure 2 shows some of the variability in the gross lesions associated with NE, and the scores to which they would be assigned.

**Table 2:** **Scoring system for experimental necrotic enteritis lesions (slightly modified from**[32]**) proposed for international adoption**

**Score**	**Lesion**	**Number of lesions**
0	No gross lesions	-
1	Thin or friable walls, or diffuse superficial but removable fibrin	-
2	Focal necrosis or ulceration, or non-removable fibrin deposit	1 to 5 foci
3	Focal necrosis or ulceration, or non-removable fibrin deposit	6 to 15 foci
4	Focal necrosis or ulceration, or non-removable fibrin deposit	16 or more foci
5	Patches of necrosis 2 to 3 cm long	Variable
6	Diffuse necrosis typical of field cases	Variable, but extensive

Although some researchers have included histopathology as part of the scoring of lesions [19,41], this is both expensive and has considerable potential for bias, since lesions are usually pre-selected for histopathological examination. Our recommendation is therefore not to include histopathology as part of a routine scoring system, unless there are special reasons to do so.

## 6. Reproduction of clinical and subclinical necrotic enteritis

Necrotic enteritis, can occur in two clinically different forms; clinical and subclinical [15]. The clinical form of the disease is associated with signs such as depression, ruffled feathers, diarrhea and even mortalities, while no clinical signs or mortality occur in the subclinical form [7,14,69,89,90]. Therefore if researchers are seeking to produce a mild form of the disease, for example to study the effect of interventions including prebiotics on production parameters over time, they may be interested to reproduce the less severe, subclinical, form of NE [41,90,91].

Factors determining outcome of the clinical form of NE with severe lesions and mortalities have been discussed; as noted, it is possible to vary parameters to determine the severity of the disease. To reproduce subclinical NE, the approach is to use approaches outlined to reduce the severity of the disease produced. For example, using even relatively large numbers of *C. perfringens* (10^8^-10^10^ CFU/mL) when challenged by oral route, just once or twice or even 3 times a day for 4–5 days), when not combined with feed factors (high protein, NSP-rich grains) or use of coccidia or IBD vaccines, produced a subclinical form of the disease with no clinical signs [19,89,90]. Using *C. perfringens* strains that lack *tpeL*, or feeding birds 24-h cultures of FTG, will predictably reduce disease severity. Nevertheless, using strains that lack *netB* will almost certainly never produce subclinical NE [79].

Figure 1 summarizes important factors affecting successful induction of NE.

## 7. Determining performance parameters (weight gain, feed intake, feed conversion ratio)

Since there is a correlation between performance parameters and lesion scores [87], measuring the performance of the birds, such as weight gain, feed intake and food conversion efficiency (FCR), can be used to evaluate the severity of the disease or the effect of interventions on disease [24-26,60]. This evaluation can be important in subclinical NE, when no clinical signs or mortalities occur. For this purpose, birds can be weighed every week, and at the time of interventions. Measuring feed consumption enables the researcher to determine the FCR. Groups will need to be appropriately large and randomized for performance parameters to be assessed for statistical significance.

## 8. Welfare considerations

Proposed studies on the reproduction of NE will require approval under national and local animal care legislation and regulations. It is important that the welfare of experimental birds be of the highest concern to researchers. An example of a score sheet and the criteria we use for humane euthanasia is given in Table 3. It should be noted that birds with NE often appear normal until shortly before they die. We euthanize birds humanely using immersion in 100% CO_2_ gas.

**Table 3:** An example of a scoring sheet used in a necrotic enteritis reproduction study

**Date and Time dd/mm/yy and am-pm**	**Cage # or Group # as appears on the cage**	**Number of birds in the cage / group**	General behaviour*: Score		**Comments**
			**0**	bright and alert;	
			**1**	reduced spontaneous activity	
			**2**	socially isolated but moves when approached	
			**3**	pronounced lethargy, only moves when stimulated	

*Actions to be taken.

During experimental infection the birds will be observed every 4 h and the last observation will be made late evening around 8 pm. Birds will be examined around 2 AM, twice on day 3 and 4 of infection (approx. 12:30 AM, 4:30 AM).

Birds showing scores of 1 will be head-marked with a marker pen. If the signs still persist 4 h later or if birds have or develop a score of 2 (or 3), such birds will be euthanized immediately using CO_2_.

## 9. Conclusions

This review has critically evaluated factors that affect the reproduction of NE in chickens. The reproduction of many infections experimentally is never guaranteed, but understanding the many variables that affect outcome and careful attention to these should allow researchers to reliably produce the disease. The use of small scale pilot studies should also identify the optimal way to produce the disease of desired severity. Best of luck!

## Competing interests

The authors declare that there is no competing interest for this review paper.

## Authors’ contributions

BS: Gathering information and papers, writing the outlines, writing the paper; JP: Proposing the subject, reviewing and editing the paper; AV: Preparation and interpretation of the pictures for the scoring system. All authors agreed on outlines and the final version of the paper.
